# A new species of *Euchaetes* Harris from southern Arizona (Erebidae, Arctiinae)

**DOI:** 10.3897/zookeys.788.26310

**Published:** 2018-10-08

**Authors:** Raymond B. Nagle, B. Christian Schmidt

**Affiliations:** 1 Department of Pathology, University of Arizona, Tucson, AZ, USA University of Arizona Tucson United States of America; 2 Canadian National Collection of Insects, Arachnids and Nematodes, Agriculture and Agri-Food Canada, K.W. Neatby Bldg., 960 Carling Ave., Ottawa, ON, Canada K1A 0C6, USA Canadian National Collection of Insects, Arachnids and Nematodes Ottawa Canada

**Keywords:** *
Euchaetes helena
*, *
Pygarctia
*, Sonoran Desert

## Abstract

*Euchaetesnancyae***sp. n.** is described from southeastern Arizona. Although superficially similar to species of *Pygarctia* Grote, structural and molecular variation shows it to be most closely related to *Euchaeteshelena* (Cassino). Adults, genitalic structure, eggs, and first instar larvae are described and illustrated. The larval host plant remains unknown. *Euchaeteshelena* is confirmed as occurring in Mexico.

## Introduction

The genus *Euchaetes* Harris currently encompasses 20 species (Vincent and Laguerre 2014; Lafontaine and Schmidt 2010), most being typical of arid and semi-arid habitats of the American Southwest and Mexico. Two species described from Brazil are not congeneric (Watson and Goodger 1986, Hendrickson 2014). There is no modern revision of the genus, although Hendrickson (2014) provides an as yet unpublished revision wherein a number of new generic combinations are proposed. As a revision will require a review of all species in the genus, in addition to the closely related *Pygoctenucha* Grote and *Pygarctia* Grote, we place the new species described herein within the current but unrevised concept of *Euchaetes*, pending an in-depth review of the *Euchaetes* group of genera. Preliminary phylogenetic analyses by DaCosta et al. (2006) and Hendrickson (2014) indicate that *Euchaetes* is polyphyletic.

## Materials and methods

Genitalic preparation techniques follow Jaeger (2017). Briefly, abdomens were macerated in 10% KOH solution overnight at room temperature, followed by cursory cleaning and separation of the genitalic capsule, and sequential transfer to 50% EtOH, 70% EtOH and 95% isopropanol. Vesica and corpus bursae inflation was carried out in 50% EtOH, followed by transfer to 70% EtOH for staining. Two stains (both in ethanol solution) were used, first chlorazol black (10 sec) then acidified eosin Y (4 + 4 sec in microwave). Stained tissues were dehydrated in 95% isopropanol before slide mounting in Euparal. Genitalia were imaged using a Leica DFC450 camera, Leica Application Suite 4.8 with a Leica M205C stereo microscope, and processed in Adobe PhotoShop. Interpretation of genitalic morphology and associated terminology differs from that of DaCosta et al. (2006) and Hendrickson (2014) in several respects. We consider the female appendix bursae in the sense of DaCosta to actually represent the bulla seminalis (see e.g., Bendib and Minet 1999), whereas the structure termed the “saccus” is the sacculus. Botanical nomenclature follows the PLANTS database (USDA 2018).

Variation of the ‘barcode’ section of the COI gene of two *Euchaetesnancyae* specimens was compared to all other known North American *Euchaetes* species (Zahiri et al. 2017). DNA extraction, PCR amplification, and sequencing of the COI barcode region was performed at the Canadian Centre for DNA Barcoding (CCDB) and followed standard protocols (Hebert et al. 2013; http://www.ccdb.ca/resources.php). DNA sequence analysis metrics were obtained from the Barcode of Life web interface. Resulting data were managed and analyzed using BOLD (Barcode of Life Data Systems; http://v4.boldsystems.org/). Mitogenomic divergence was calculated based on Kimura 2-Parameter (K2P) distances of COI barcodes.

Repository abbreviations are as follows:

**AMNH** American Museum of Natural History, New York, NY

**CNC** Canadian National Collection of Insects, Arachnids and Nematodes, Ottawa, ON

**USNM** National Museum of Natural History (formerly United States National Museum), Washington, DC

**RBN** Raymond B. Nagle Collection, Tucson, AZ

**EJR** Evan J. Rand Collection, Phoenix, AZ

## Results and discussion

Examination of the type specimens of *Euchaetes* and *Pygarctia* described from southwestern USA and Mexico (USNM, AMNH and as illustrated in Hendrickson 2014), in addition to comparison of museum material, shows that the species described here does not have any close relatives in either genus. The lack of a prothoracic foretibial claw, and long second segment of the labial palpus places the new species in *Euchaetes* rather than *Pygarctia* (Forbes 1960; DaCosta et al. 2006: fig. 3). Structural similarities and DNA barcode data associate this new species with *Euchaeteshelena* (Cassino), *E.zella* (Dyar) and *E.fusca* (Rothschild), as discussed below.

### Euchaetes nancyae sp. n.

Taxon classificationAnimaliaLepidopteraErebidae

http://zoobank.org/BA87F037-DB12-4D3D-A76F-B8294D0F1D63

**Figures 1–6.**
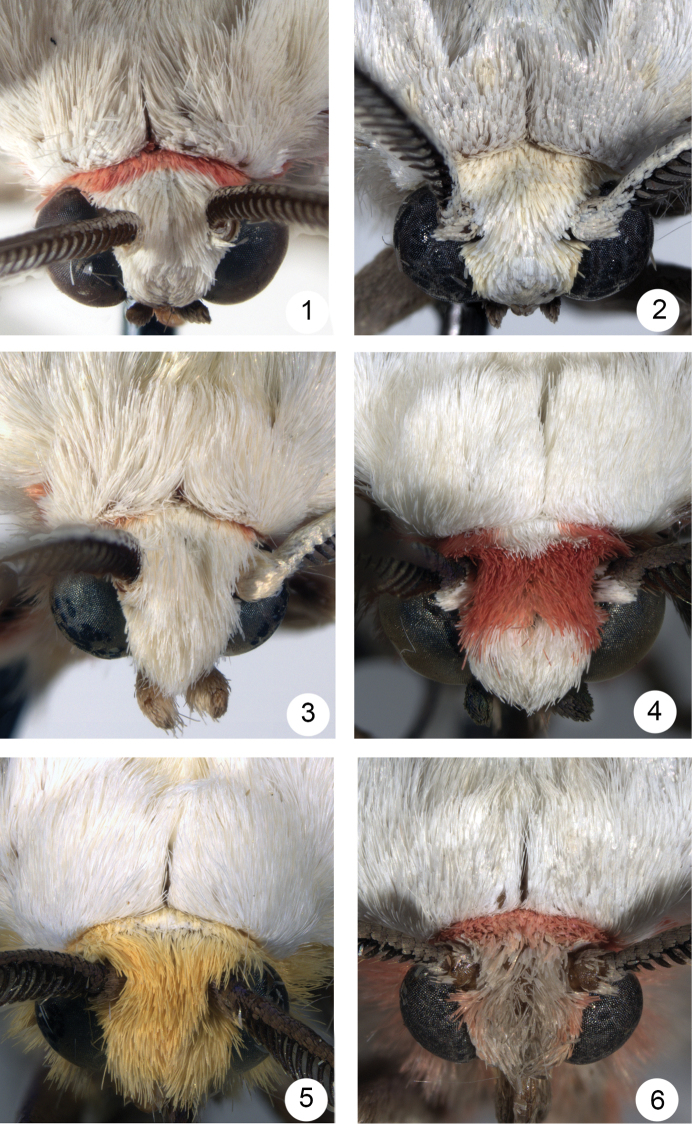
Head and thoracic colour patterns of *Euchaetesnancyae* and similar species. **1***Euchaetesnancyae***2***Euchaeteshelena***3***Euchaetescastalla***4***Pygarctiaroseicapitis***5***Pygarctiaflavidorsalis***6***Pygarctialorula*.

**Figures 7, 8.**
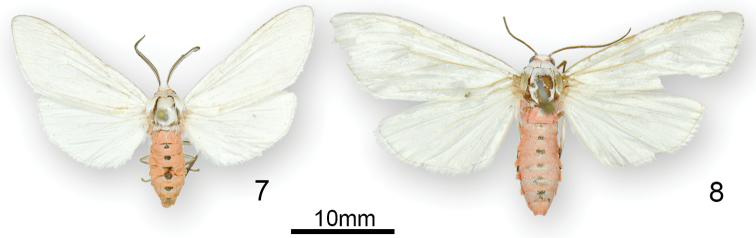
Adults of *Euchaetesnancyae* male (**7**) and female (**8**).

**Figures 9, 10.**
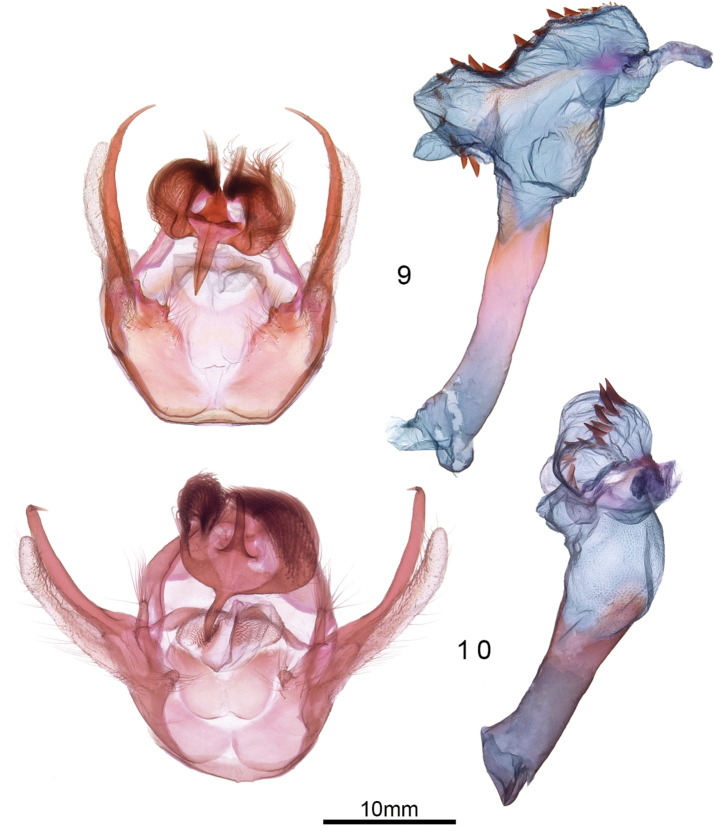
Male genitalia of **9***Euchaetesnancyae* and **10***E.helena*.

**Figures 11, 12.**
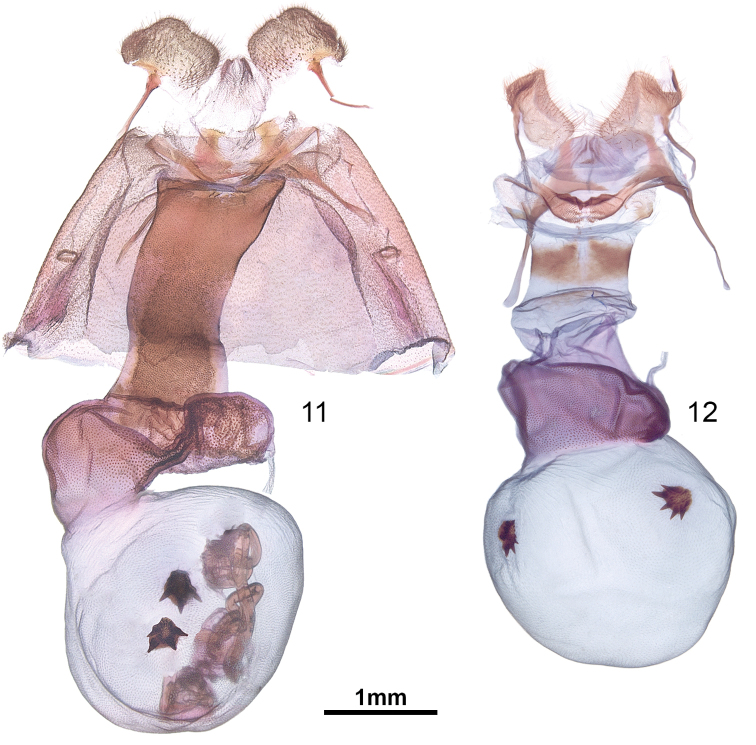
Female genitalia of **11***Euchaetesnancyae* and **12***E.helena*.

Figures 1 , 7–8 , 9 , 11

#### Type material.

**Holotype** ♂. Arizona: Santa Cruz Co., Peña Blanca Canyon, 4000', 22–23 Jul 1999, J. B. Walsh; CNCLEP 79872 [CNC]. **Paratypes** 7♂ 5♀. **Arizona**: Santa Cruz Co., 4 mi W Peña Blanca Lake, 20 Jul 2000, R.B. Nagle, 1♂; CNC dissection # 17027, [CNC]; Santa Cruz Co., Hall Ranch, 31.60°N 110.73°W, [7 km NNE Patagonia], 27 Aug 2016, R. B. Nagle 1♀, CNC dissection # CNC17658, DNA voucher # 16-132 [CNC]; same data as previous, 1♀, dissection # RBN001, DNA voucher # 16-133 [RBN]; same data as previous, 1♂, [RBN]; Santa Cruz Co., Patagonia roadside rest stop, mile marker 15.6, 5.Jul.2004, B. Walsh, 1♀ [RBN]; Santa Cruz Co., California Gulch, 31°25'18.3"N, 111°14'40.02"W, 21.Jul.2012, E. J. Rand, 8♂ 2♀, [EJR].

#### Etymology.

*Euchaetesnancyae* is named in honor of the senior author’s wife who not only has long supported her husband’s study of southwest moths but has also played host to numerous visiting lepidopterists. Nancy won a weekend trip to the Hall Ranch, which led to the initial capture and discovery of this species.

#### Diagnosis.

*Euchaetesnancyae* is superficially similar to other species of *Euchaetes* and *Pygarctia* that have predominantly white or grey-white wings, but can generally be distinguished by examination of the head and thoracic colour patterns alone, as illustrated in Figs 1–6. *Euchaeteshelena* and *Pygarctiaflavidorsalis* Barnes & McDunnough have yellow instead of pink scaling on the head. The most common look-alike species that is sympatric with *E.nancyae* is *Pygarctiaroseicapitis* (Neumögen & Dyar), but *E.nancyae* differs most obviously from that species by the white (Figure 1) rather than pink (Figure 4) vertex of the head. Wing and thoracic colour of *E.nancyae* is also similar to those of *E.castalla* Barnes & McDunnough; however, *E.castalla* has less pink scaling along the posterior margin of the head that is interrupted medially with white; the pink scales are often not visible without magnification and give the prothoracic collar the appearance of being entirely white (Figure 3); females of *E.castalla* have a white abdominal tuft, whereas the tuft is absent in *E.nancyae*. The distributions of *E.nancyae* and *E.castalla* overlap in at least southeastern Arizona (e.g. California Gulch, Pima Co.), and may do so also in northern Mexico. Morphologically, the male uncus and vesica of *E.nancyae* is highly distinctive, particularly the setation of the lateral lobes and the structure of the dorsomedial lobe, as described below and illustrated in Figs 9, 10. Females differ from those of *E.helena* in the shape of the cervix bursae and the papillae anales (Figs 11, 12). Structurally, *E.nancyae* differs from all *Pygarctia* species by the lack of a foretibial spine and a shorter 2^nd^ segment of the labial palpus, in addition to the genus traits given below in the Discussion.

DNA barcode sequences of *E.nancyae* are more than 6% divergent from all other North American *Euchaetes*, in addition to several undescribed Mexican and Central American species. The two sequenced specimens of *E.nancyae* differ by 0.46%, forming a unique BIN (Barcode Index Number; Rathnasingham and Hebert 2013). The most similar species based on nearest-neighbour distance analysis is *Euchaeteshelena*, differing by a minimum of 6.61%.

#### Description.

*Head*. Vestiture of sexes similar; frons and vertex covered with white scales; posterior margin of head with a delicate ring or collar of pale orange-pink scales, extending around posterior and ventral margin of eye; male antenna bipectinate, longest rami 2.5 x longer than antennal segment, scape and proximal third dorsally scaled with white, grading into darker pale grey-brown scaling over distal two-thirds; female antenna biserrate, dorsal vestiture similar to that of male. First segment of labial palpus with relatively long, pale orange-pink scales along ventral edge, second segment covered with white scales, third segment covered with light brown scales; length ratio of segments 1:2:3 = 2.2 : 3.0 : 1.0.

*Thorax*. Vestiture of sexes similar; patagium, tegula, and thorax covered with greyish-white scales, lateral margin of patagium and tegula with a border of long pale pink hairs extending underneath wings; foreleg coxa pale orange pink with a medial patch of pale grey-brown scales; foretibia without claw at apex; femur and tibia white dorsally, ventrally a mix of white and pale grey-brown scales, tibial spurs and tarsal segments pale grey brown; mid and hind legs with coxa, femur, and tibia covered with a mix of white and pale grey brown scales; metepisternum lacking microtymbals.

*Forewing and hindwing*. Dorsally both wings and fringe entirely silvery greyish white, lacking any distinguishable markings in both sexes; ventrally, forewing a slightly darker shade of greyish white than hindwing. Male forewing length 15.2–15.8 mm (n = 2), female 18.6–19.5 mm (n = 2).

*Abdomen.* Colouration of sexes similar; dorsum pale silvery pinkish orange with a row of 6–7 dorsomedial macules at anterior tergal margin, this dark grey and indistinctly ringed with whitish grey; laterally with a row of prominent black maculae at anterolateral angle; ventrum silvery whitish grey; female without terminal tuft of long scales; intersegmental membrane between sternites 7–8 of male with well-developed coremata, nearly length of abdomen when fully extended.

*Male genitalia*. Gross morphology typical of *Euchaetes*, with costa and sacullus deeply divided, costa forming a long prong-like finger, and sacculus forming a membranous, elongate lobe; saccus broad and collar like, not tapered to a point medio-ventrally as in many arctiines; juxta lightly sclerotized and indistinctly differentiated, broadly U-shaped; uncus complex, with a dorsoventrally flattened lateral lobe; each lobe densely setose, forming an evenly sinuate “hair-do” that terminates in an anteriorly-directed, dense, pointed tuft; dorsomedial process of uncus with a pyramidal base, terminating in a laterally flattened crest; apex of uncus laterally flattened and shaped like a broad bird’s beak in lateral view; phallus 5 × longer than wide, curved slightly dorsad; vesica roughly kidney shaped with small subbasal diverticulum; disto-medial row of approximately 20 sawtooth-like cornuti, decreasing in size towards ductus ejaculatorius.

*Female genitalia.* Distal margin of papillae anales irregular rounded quadrate; papillae anales moderately setose, densely and finely setose dorsally below apertures of dorsal pheromone gland; pseudopapillae anales membranous; dorsal pheromone gland unbranched, terminating as pair of openings; length of anterior and posterior apophyses approximately equal to maximum width of papillae; cervix bursae moderately sclerotized, internally rugose near junction with ductus, remainder with regularly spaced microtrichia or spicules; ductus bursa flattened dorsoventrally and ribbon-like; corpus bursa essentially spherical, with two star-like signa, one dorsal and one ventral; ventral signum with four arms or rays, dorsal one with three.

#### Biology and distribution.

The larval hostplants of *E.nancyae* are unknown. Two females oviposited approximately 30 shiny yellow-white eggs measuring 0.8 mm in diameter. *Euchaetesnancyae* lacks female abdominal tufts, and eggs were not covered with abdominal setae. Presumably, all *Euchaetes* species that possess female abdominal tufts cover their eggs, such as *E.egle* (Drury). The first instar larvae were offered several species of Asclepiadaceae including *Funastrumcynanchoides* (Decne.) Schltr. and one species of Euphorbiaceae, *Chamaesycehyssopifolia* (L.), but refused to feed and perished. The related species *Euchaeteszella* and *E.perlevis* feed on *Funastrumcynanchoides*, and *Euchaetesfusca* larvae feed on the *Cnidoscolusangustidens* Torr. (Euphorbiaceae). The larval foodplant of the probable sister species *Euchaeteshelena* from Texas is also unknown, but larvae of that species also do not accept Asclepiadaceae (D. Wagner, pers. comm.).

*Euchaetesnancyae* is known only from the type series, collected in Sonoran desert habitat along the Mexican border in Santa Cruz County, Arizona. It undoubtedly also occurs in adjacent parts of Mexico. *Euchaeteshelena* occurs in Texas (Davis Mountains), and was listed as likely occurring in Mexico by Vincent and Laguerre (2014); we here confirm the occurrence of *E.helena* from Coahuila, verified by DNA barcode sequence (DNA vouchers CNCLEP 00113425 and CNCLEP00113424).

## Conclusion

Based on morphological similarities, *Euchaetesnancyae* is most closely related to *E.helena*, *E.zella*, *E.perlevis* and *E.fusca*. Species in this group all share 1) an untufted female abdomen (tufted in other North American species), 2) a well-developed male abdominal coremata, and 3) lack metepisternal microtymbals (the “Striate Band” of Forbes 1960). With the addition of *E.cressida* (Dyar), this assemblage corresponds to “*Euchaetes* Clade 2” of DaCosta et al. (2006). Despite the similarities of *Euchaetesnancyae* to this group, *E.nancyae* represents a surprisingly divergent evolutionary lineage within *Euchaetes*, and should help resolve the phylogeny of this complex group of genera.

## Supplementary Material

XML Treatment for Euchaetes nancyae
